# Rec-DCM-Eigen: Reconstructing a Less Parsimonious but More Accurate Tree in Shorter Time

**DOI:** 10.1371/journal.pone.0022483

**Published:** 2011-08-24

**Authors:** Seunghwa Kang, Jijun Tang, Stephen W. Schaeffer, David A. Bader

**Affiliations:** 1 School of Electrical and Computer Engineering, Georgia Institute of Technology, Atlanta, Georgia, United States of America; 2 Department of Computer Science and Engineering, University of South Carolina, Columbia, South Carolina, United States of America; 3 Department of Biology, The Pennsylvania State University, University Park, Pennsylvania, United States of America; 4 School of Computational Science and Engineering, Georgia Institute of Technology, Atlanta, Georgia, United States of America; Kyushu Institute of Technology, Japan

## Abstract

Maximum parsimony (MP) methods aim to reconstruct the phylogeny of extant species by finding the most parsimonious evolutionary scenario using the species' genome data. MP methods are considered to be accurate, but they are also computationally expensive especially for a large number of species. Several disk-covering methods (DCMs), which decompose the input species to multiple overlapping subgroups (or disks), have been proposed to solve the problem in a divide-and-conquer way.

We design a new DCM based on the spectral method and also develop the *COGNAC* (Comparing Orders of Genes using Novel Algorithms and high-performance Computers) software package. *COGNAC* uses the new DCM to reduce the phylogenetic tree search space and selects an output tree from the reduced search space based on the MP principle. We test the new DCM using gene order data and inversion distance. The new DCM not only reduces the number of candidate tree topologies but also excludes erroneous tree topologies which can be selected by original MP methods. Initial labeling of internal genomes affects the accuracy of MP methods using gene order data, and the new DCM enables more accurate initial labeling as well. *COGNAC* demonstrates superior accuracy as a consequence. We compare *COGNAC* with FastME and the combination of the state of the art DCM (Rec-I-DCM3) and GRAPPA . *COGNAC* clearly outperforms FastME in accuracy. *COGNAC* –using the new DCM–also reconstructs a much more accurate tree in significantly shorter time than GRAPPA with Rec-I-DCM3.

## Introduction

Maximum parsimony (MP) [1], [2] methods enumerate candidate trees for the input species and select the most parsimonious tree as an output tree by processing the input species' genome data (such as nucleotide sequence data or gene order data). MP methods are considered to be accurate, but finding the most parsimonious tree is often computationally very expensive. Even with an efficient branch and bounding strategy and for a relatively small number of species, MP methods need to evaluate a large number of candidate tree topologies. The number becomes prohibitively large for many species.

Ranking different tree topologies is much more expensive for gene order data than nucleotide sequence data. For gene order data, there is no known algorithm to find the most parsimonious labeling of the internal genomes in a tree to compute the tree's parsimony score if the tree has more than three leaf genomes [3]. Even heuristics [4] to score a topology are NP-hard assuming a linear-time computable pairwise distance metric (*e.g.* breakpoint distance, inversion distance, and DCJ distance). Computing the distance between two genomes can be NP-hard as well based on the definition of distance [4]. A large number of candidate trees is even more problematic for gene order data as a result.

Warnow and her group [5]–[7] have proposed several disk-covering methods (DCMs) to reduce the candidate tree search space. DCMs decompose the input genomes to multiple overlapping disks; find a tree topology for each disk; and merge the topologies to reconstruct a tree for the entire set of input genomes. Rec-I-DCM3 [7], which is the most recently published DCM, recursively decomposes the input genomes to further reduce the search space and iterates the process multiple times to refine the reconstructed tree. However, existing DCMs have several shortcomings. Computing a disk decomposition is expensive for the original DCM [5] and DCM2 [6]. Rec-I-DCM3 computes a decomposition faster but requires multiple iterations to achieve high accuracy. The existing DCMs place a significant number of genomes in the overlapping region, and this also increases computing time.

This paper presents a new DCM that improves on existing techniques. We observe that phylogenetic tree reconstruction problems resemble graph bi-partitioning and clustering problems. Every edge in a phylogenetic tree bi-partitions the leaf genomes in the tree. A phylogenetic tree often includes multiple sets of close genomes–or clusters–as well. The spectral method [8], [9], followed by heuristics to refine the initial result, is one of the most successful methods in solving graph bi-partitioning and clustering problems.

The new DCM is based on the spectral method and uses the pairwise distances between leaf genomes to find a disk decomposition. The spectral method computes the second smallest eigenvalue and an 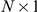 eigenvector for the eigenvalue assuming 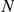 input genomes. The method initially places a genome in one partition or the other based on the sign of the corresponding eigenvector element. The magnitude of the vector element indicates the confidence level of the decision, and the new DCM uses this information to refine the initial bi-partitioning.

The MP principle is effective in many cases due to the strong correlation between the parsimony score of a tree topology and the topology's accuracy. However, the most parsimonious tree is not always the most accurate tree. Long branch attraction (LBA) [10], [11] is one circumstance in which trees that seem more parsimonious can be less accurate. LBA becomes significant if a tree that best describes the phylogenetic relationships of the input species has a mix of long and short edges; if we can bi-partition the input species over a long edge in the tree, this can reduce the impact of LBA in reconstructing a sub-topology for a subset of the input genomes. If there is no long uninterrupted edge in the evolutionary history of the input species, finding an overlapping decomposition is better for accurate reconstruction. The gap between the minimum positive eigenvector element and the maximum negative eigenvector element is large if there is a long uninterrupted edge in a tree that best depicts the evolutionary history, and the new DCM exploits this information to find a close to ideal decomposition.

A new computer program, *COGNAC* (**C**omparing **O**rders of **G**enes using **N**ovel **A**lgorithms and high-performance **C**omputers), recursively decomposes a disk using the new DCM till three or fewer genomes are left and builds a binary disk tree. After building a binary disk tree, *COGNAC* , based on the MP principle, reconstructs sub-topologies of the leaf disks in the disk tree and recursively merges the sub-topologies to reconstruct a tree for the complete set of input genomes.

*COGNAC* uses the pairwise distances and the new DCM to reduce the candidate tree search space and uses the GRAPPA method [12] (which is based on the MP principle) to select an output tree from the reduced set of candidate tree topologies. This approach enables high accuracy in addition to fast execution time. The new DCM not only reduces the number of candidate tree topologies but also excludes erroneous tree topologies that can result from original MP methods. Initial labeling of internal genomes affects the accuracy of MP methods using gene order data, and the new DCM enables accurate initial labeling as well. We provide two examples to illustrate this in this paper using gene order data and inversion distance.

To experimentally evaluate *COGNAC* , we use simulators ([13], [14], and [15]) to generate model trees and apply inversion mutations based on the model trees to generate test data. We first compare *COGNAC* with FastME . We use GRAPPA 's EDE [16] to correct the pairwise distances for multiple mutation events and provide the corrected distance matrix as the FastME input data. We also compare *COGNAC* with the combination of Rec-I-DCM3 , the state of the art DCM, and GRAPPA [12] which is known to be one of the most accurate methods for phylogenetic tree reconstruction using gene order data. *COGNAC* clearly outperforms FastME in accuracy though *COGNAC* , in general, runs slower than FastME . However, considering that *COGNAC* is an MP method for gene order data and FastME is a distance method, the gap in execution time is significantly smaller than typical cases. We also use the combination of Rec-I-DCM3 and GRAPPA using the FastME output tree as a guide tree, but this approach returns less accurate trees than even the FastME output tree and runs multiple orders of magnitude slower than *COGNAC* . *COGNAC* and data sets used in this paper are freely available from http://code.google.com/p/cognac in accordance with a report from an NSF funded software sustainability and reusability workshop [17].

## Methods

### Gene Order Data, Genome Rearrangement, Inversion Distance, GRAPPA, Median Problem, and Distance Correction

Assume a set of 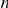 genes (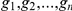) common to all the input genomes. Gene order data represent each input genome as a sequence of the 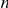 genes. A gene in gene order data has a sign (either 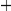 or 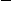) based on the gene's strandedness. Genome rearrangement events reorder genes in gene order data and also change the strandedness of genes [18], [19]. Inversion mutations are one of the genome rearrangement events that reorganize the order of genes, and many organellar genomes as well as eukaryotic lineages ([18]–[20]) evolve with inversion as a main evolutionary mechanism. An inversion reverses the order and the sign of genes. Assume a genome (say 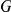) represented by the following order of genes: 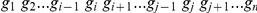. An inversion between the 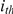 and the 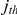 genes transforms 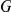 to 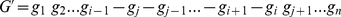. The length of an inversion is the number of genes involved in the inversion event (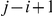 in the example). The inversion distance between two genomes is the minimum number of inversions required to transform one genome to the other. A phylogenetic tree reconstructed using inversion distance is often more accurate than a tree reconstructed using breakpoint distance [21]. We refer readers to [4] and [22] for further details about gene order data and genome rearrangement events.

GRAPPA [12], along with MGR [23], is the most accurate and widely used software for reconstructing a phylogenetic tree using gene order data. GRAPPA supports inversion distance as well. GRAPPA exhaustively searches the entire candidate tree space (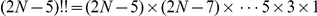 unrooted tree topologies for 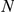 genomes); enumerates candidate tree topologies with the branch-and-bound technique; scores the enumerated candidate tree topologies; and selects the trees with the lowest parsimony score. To score a tree topology, GRAPPA repeatedly solves median problems till the score of the topology converges. A median problem finds a median genome of three genomes, and a median genome of three genomes is a genome that minimizes the sum of distances between the genome and the three input genomes.

The inversion distance between two genomes saturates if the true number of inversions between the two genomes exceeds a certain threshold value–for two genomes with 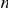 genes, the inversion distance between the two genomes cannot exceed 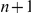. This lowers the accuracy of reconstructed trees. Common adjacencies of genes in two genomes saturate significantly later than the inversion distance between the two genomes, and GRAPPA 's EDE [16] exploits this information to estimate the true number of inversions between two genomes even after the inversion distance between the two genomes saturates.

### The Spectral Method

The spectral method has been widely used in solving graph bi-partitioning [8] and clustering [9] problems. Assume a graph with vertices and edges–each vertex has some computation and the weight of an edge is proportional to the amount of communication between the two vertices connected by the edge. To process the graph using a parallel computer by distributing vertices (and associated computations) to multiple computing nodes, it is important to balance the amount of computing in each computing node while minimizing the communication between two different computing nodes. Graph bi-partitioning aims to satisfy the requirement by finding a bi-partition that minimizes the sum of the edge weights between two partitions (the sum corresponds to the amount of communication) while balancing the number of vertices in each partition. The goal of clustering is to partition the input data points to multiple highly similar subgroups. Assume another graph with vertices and edges–each vertex represents a data point and an edge weight is proportional to the similarity between the two vertices. To solve a clustering problem, one needs to partition the graph with two (or more) subgroups with a large sum of the edge weights within a subgroup (to maximize the similarity) while minimizing the sum of the edge weights between two different subgroups (to minimize the similarity). The spectral method has been very successful in finding an initial solution for these problems, and we apply the method to decompose the input genomes. We briefly explain the spectral method first before presenting our algorithm in detail.

The spectral method first constructs a Laplacian matrix; computes the second smallest eigenvalue and an eigenvector for the eigenvalue; and bi-partitions the input data points using the eigenvector. Assume the weighted adjacency matrix 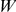, where 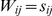 (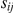 is the similarity value between the 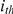 and the 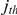 input data points, and the similarity value can be computed using the distance between the two data points). Let 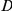 denotes the diagonal degree matrix (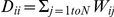) as well. Then there are several ways to construct a Laplacian matrix using 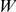 and 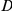, and 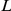 (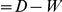), 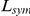 (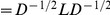), and 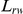 (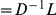) are widely used Laplacian matrices. 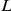 is commonly used to solve graph bi-partitioning problems, while 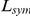 or 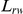 can work better for clustering problems [9]; note that 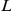 maximizes the dissimilarity between two partitions while 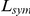 and 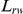 maximizes both the similarity within a partition and the dissimilarity between different partitions [9]. An eigenvalue (say 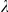) and an eigenvector (say 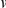) of matrix 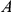 satisfies 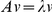. To bi-partition the input genomes, the spectral method finds the second smallest eigenvalue of the Laplacian matrix and an eigenvector for the eigenvalue. The spectral method bi-partitions the input data points using the sign of the eigenvector elements; the 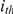 element of the eigenvector determines whether the 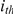 data point belongs to one partition or the other. The magnitude of an eigenvector element provides the level of confidence for the decision and a larger magnitude suggests a higher level of confidence. This information is especially valuable in finding an overlapping decomposition of the input genomes in solving our problem; we place the genomes with a small magnitude eigenvector element in the overlapping region–the specifics will be presented later in this paper. This bi-partitioning algorithm is commonly used for graph partitioning. k-means algorithm is often used instead of using the sign of eigenvector elements for clustering problems [9]. Clustering problems often partitions the input data points to more than two groups, and in this case, they use the third smallest (and larger) eigenvalues and corresponding eigenvectors in addition to an eigenvector for the second smallest eigenvalue. Our experiments show that partitioning the input genomes to more than two groups is less accurate than bi-partitioning the input genomes; thus, we focus on bi-partitioning the input genomes in this paper.

### A High-level Overview of Rec-DCM-Eigen

This section provides a high-level informal overview of the new discovering method, Rec-DCM-Eigen, before providing an in-depth description of the algorithm. There are 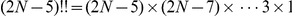 possible (unrooted) topologies for 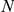 input genomes. The number grows rapidly with an increasing number of genomes or in other words, the number decreases rapidly with a decreasing number of genomes. Disk-covering methods aim to find a tree by first decomposing the entire set of input genomes to multiple subgroups; fixing a topology for each subgroup (note that the number of possible topologies for each subgroup with a smaller number genomes is much smaller than the number of possible topologies for the entire set of input genomes); and merging the topologies for the subgroups. We can apply this process recursively to further reduce the tree topology search space.

The effectiveness of disk-covering methods highly depends on the quality of disk decompositions. Several existing researches show that we can find an accurate tree if we can find the bi-partition over a long uninterrupted edge in the evolutionary history–see the next section for additional details. The two groups of genomes separated by a long uninterrupted edge are highly dissimilar–assuming that the similarity of two genomes is inversely proportional to the evolutionary distance between the two genomes. If we construct a fully connected graph with its vertices representing genomes and an edge weight representing the similarity between the two genomes connected by the edge, the spectral method is effective in finding a bi-partition with the high dissimilarity between two partitions; this coincides with the bi-partition over a long uninterrupted edge. However, there may not exist a long uninterrupted edge in the evolutionary history. The magnitude of eigenvector elements, which represents the confidence level of the bi-partitioning decision, becomes useful in this case. We bi-partition if the magnitude of every eigenvector element is large. If there are multiple eigenvector elements with a small magnitude value, we bi-partition excluding the genomes corresponding to the small magnitude eigenvector elements first and places the genomes with a low level of confidence in the overlapping region.

**Figure 1:**
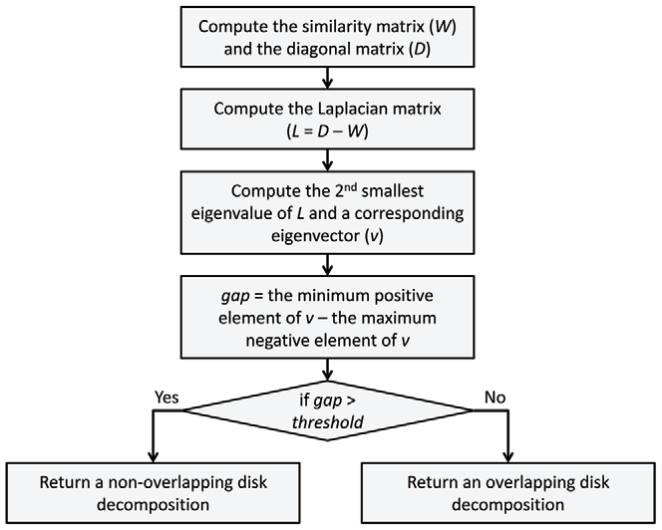
An overview of the new DCM.

Recursively applying this process largely reduces the tree topology search space. This can also increase the accuracy of a reconstruction method if the tree topology search space excludes erroneous tree topologies which can be selected by suboptimal methods. The newly designed disk-covering method, Rec-DCM-Eigen, achieves this goal. Figure 1 depicts our new DCM in high-level and the remainder of this section presents details of the algorithm.

### A New Disk-Covering Method Based on the Spectral Method

**Figure 2:**
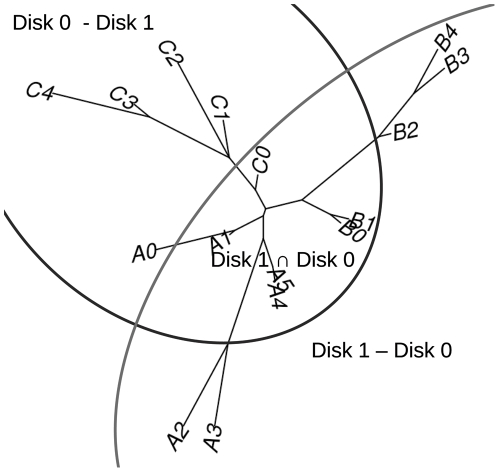
An incompatible but recoverable disk decomposition.

We define compatible, (incompatible but) recoverable, and irrecoverable disk decompositions first before presenting our new DCM. A disk decomposition is compatible if there was a speciation event in the evolutionary history (or if there is an edge in the model tree used for the simulation) that separates the genomes in the exclusive region of one disk from the genomes in the exclusive region of the other disk. Genomes in the overlapping region do not affect the compatibility. If a disk decomposition is incompatible but the incompatibility can be recovered in the merging step, then the disk decomposition is recoverable. See Figure 2 for an example.

In Figure 2, there is no edge separating A0, C1, C2, C3, and C4 from A2, A3, B2, B3, and B4; the decomposition is incompatible. Yet, if we reconstruct the correct sub-topology for each disk, we can reconstruct the correct topology for the entire set of genomes by merging the two sub-topologies without changing the sub-topologies; the decomposition is recoverable. If a decomposition is incompatible and is not recoverable, then the decomposition is irrecoverable. Our new DCM decomposes the input genomes to two disks (each disk contains a subset of the input genomes) and aims to find a compatible or at least recoverable disk decomposition.

If we can find the bi-partition over a long uninterrupted edge in the evolutionary history, this improves the accuracy of a reconstructed tree while reducing the tree topology search space. Atteson [24] proved that even the computationally inexpensive neighbor-joining (NJ) method [25] reconstructs a tree with 100% topological accuracy if the maximum gap between the additive distance (the sum of the edge lengths in the path between two genomes in a tree that best captures the evolutionary history) and the pairwise distance between two genomes in the input genomes is smaller than one half of the shortest edge in the tree to reconstruct. The gap is nearly zero for close genomes and we can reconstruct a tree with 100% topological accuracy for close genomes unless there is a very short edge in the tree. Bi-partitioning over a long edge significantly reduces the maximum pairwise distance between the genomes in each partition. Bi-partitioning over a long edge also prevents the long edge from disturbing the accurate reconstruction of the phylogeny for the genomes in the two subsets in each side of the edge [11], [26], [27]. If there is a long uninterrupted edge in the evolutionary history, detecting such a signal and finding the bi-partition over the long edge is the second goal of our new DCM.

A good disk decomposition method needs to decompose the input genomes even when there is no long uninterrupted edge in the evolutionary history if one wishes to reconstruct a phylogenetic tree for a large number of species. If there is no long edge in the evolutionary history, however, finding a bi-partition can be error prone. Bi-partitioning over a short edge often creates a longer edge which can negatively impact the accuracy of the tree as well. In this case, placing several genomes near the partition boundary in both disks improves the accuracy of a reconstructed tree. Our new DCM aims to include genomes that are necessary for the accurate reconstruction of the phylogeny of the genomes in the exclusive regions.

#### Constructing a Laplacian matrix for our DCM

There are two questions to be answered when constructing a Laplacian matrix. The first question is about defining a similarity metric. The second question is what type of a Laplacian matrix to use. One simple way to define a similarity metric is using the inverse of a pairwise distance. von Luxburg [9] suggested using the Gaussian similarity function instead (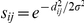, where 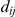 is the pairwise distance between the 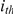 and the 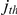 input genomes). Using just the inverse of 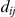 is problematic as most similarity values can have only very small values or only very large values based on the distribution of the pairwise distances. We can control the distribution of similarity values by changing 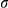 for the Gaussian similarity function, and we adopt the function and set 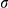 to 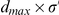 (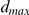 is the maximum pairwise distance between any two genomes in a disk to decompose, and we set 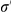 to 0.125 in our experiments).

**Figure 3:**
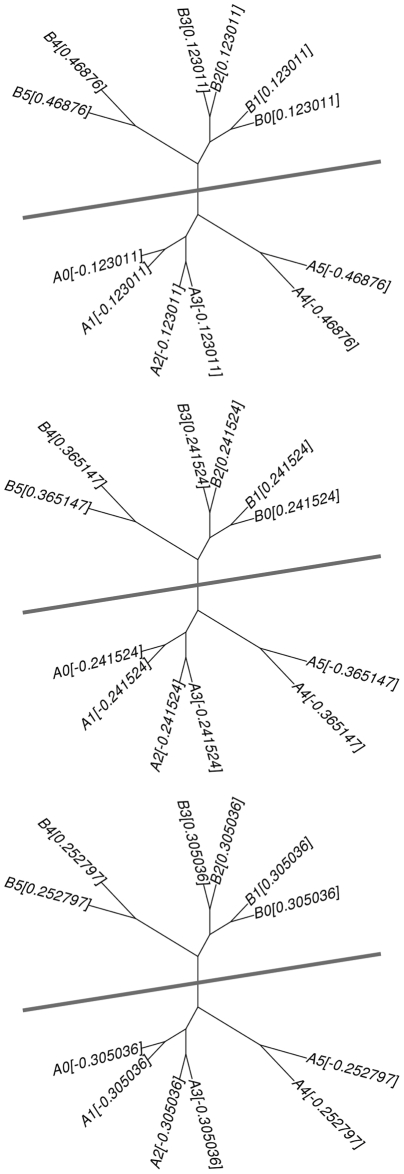
An example showing an advantage of using 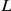 and 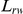 over 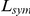. The trees above show the phylogenetic relationships of twelve genomes. The value next to a genome name is the genome's eigenvector element computed using 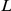 (top), 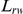 (middle), and 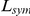 (bottom), respectively.

There are several types of Laplacian matrices such as 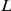, 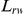, and 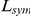. 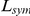 is inappropriate. von Luxburg [9] showed that if 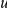 is an eigenvector of 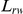 with eigenvalue 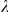 then 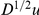 is an eigenvector of 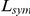 for the same eigenvalue. In other words, the eigenvector element for a genome with many close neighboring genomes has a larger magnitude with 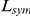 than 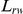. This is undesirable for our purpose because genomes near the partition boundary should have a lower magnitude regardless of whether there are similar genomes or not. Figure 3 illustrates the point.

In the figure, 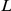, 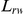, and 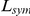 suggest the same bi-partitioning if we bi-partition the genomes based on the sign of eigenvector elements. 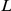 and 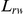 also assign small magnitude values to the genomes near the partition boundary, but 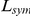 assigns large magnitude values to the genomes near the partition boundary as the genomes near the boundary has many close neighbors. As we use the magnitude of the eigenvector element to decide whether to place a genome in the overlapping region or not, this is undesirable; we reject 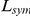.

**Figure 4:**
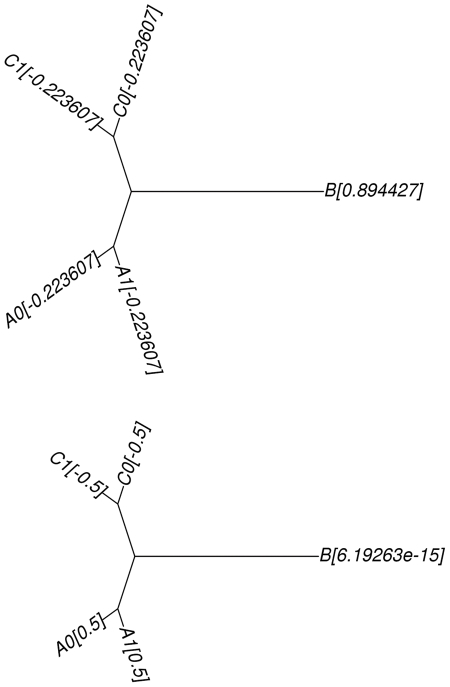
An example showing an advantage of using 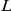 over 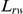. The trees above show the phylogenetic relationships of five genomes. The value next to a genome name is the genome's eigenvector element computed using 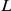 (top) and 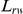 (bottom), respectively.

We discard 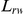 next. 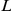 focuses on minimizing the dissimilarity between two partitions while 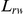 attempts to maximize the similarity within a partition as well [9]. 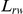 is more desirable for many clustering problems as maximizing the similarity within a cluster is an important objective of those problems. The key objective of our disk decomposition problem is not maximizing the similarity within a partition but finding the bi-partition over a long edge. 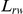 often returns an inferior decomposition than 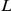 in this perspective. Figure 4 provides an example.

In the figure, 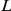 suggests the bi-partition over the longest edge while 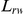 suggests the disk decomposition that places A0 and A1 in one partition and C0 and C1 in the other. B has a very small magnitude and will be included in both disks. 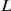 suggests a better bi-partition for our purpose, and we use 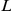 in our experiments.

The perturbation theory also supports the use of 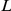 (and 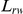) over 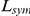. The theory states that for a perturbed matrix (say 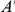 = 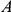
+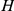, 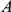 is a noise free matrix and 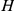 is a noise matrix), the error due to 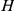 in computing an eigenvector is not significant unless the norm of 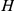 is very large for a Laplacian matrix [9]. This is especially true for 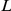 and 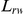 but this does not hold for 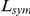 if there are low degree vertices [9]. In our problem, the non-additivity of the pairwise distance between two genomes corresponds to noise as the gap (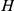) between the ideal Laplacian matrix constructed using additive distances (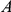, note that we can construct a 100% accurate topology if we can find the additive pairwise distances between the input genomes [24]) and a Laplacian matrix constructed using computed distances (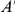) increases as the pairwise distances between the input genomes become more non-additive. If we use inversion distance but the input genomes evolve via a mix of inversions and transpositions, this also increases the level of noise. 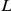 is robust to such noise, and this also justifies the use of 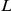 over 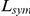.

#### Finding a disk decomposition using an eigenvector

The spectral method provides a reasonable initial solution in solving graph bi-partitioning and clustering problems, but the method often requires a refinement step to find a higher quality solution for such problems [28], [29]. In the context of disk decomposition for phylogenetic tree reconstruction, a refinement step is necessary if there is no long edge in a tree that best captures the phylogenetic relationships of the input species. The spectral method suggests a bi-partition that has high dissimilarity between the two resulting partitions. If there is a long uninterrupted edge in the evolutionary history, the two groups of genomes in each side of the edge are highly dissimilar, and the spectral method is effective in finding the bi-partition over such a long edge. If there is no long uninterrupted edge, it is often very difficult to bi-partition the leaf genomes to two highly dissimilar subsets. In this case, bi-partitioning the input genomes based on the sign of an eigenvector element can often lead to an incompatible disk decomposition as the spectral method bi-partitions based on the weak dissimilarity signal in the input genomes. However, if we place genomes near the partition boundary in the overlapping region, the error can be recovered. An eigenvector element of a genome near the partition boundary has a small magnitude. Our DCM exploits this. It sorts the input genomes using their eigenvector element as a key; finds an initial bi-partition by placing the genomes with a negative eigenvector element in one disk and the genomes with a positive eigenvector element in the other; computes the difference between the smallest positive eigenvector element and the largest negative eigenvector element; places the genomes with a small magnitude eigenvector element in the overlapping region if the gap is smaller than a certain threshold value (say 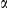). The algorithm visits genomes in each partition starting from the partition boundary in the sorted list until the gap between a current genome and the next genome exceeds another threshold value (say 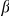). All the visited genomes are placed in the overlapping region. Using 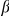 prevents placing only a subset of very close genomes in one partition. We use Intel MKL as an eigensolver. Intel MKL returns a normalized eigenvector, and 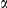 and 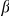 also need to be scaled based on the number of genomes in a disk to partition. For a disk with 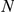 genomes, we use 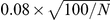 for 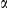 and 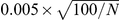 for 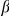, respectively.

**Figure 5:**
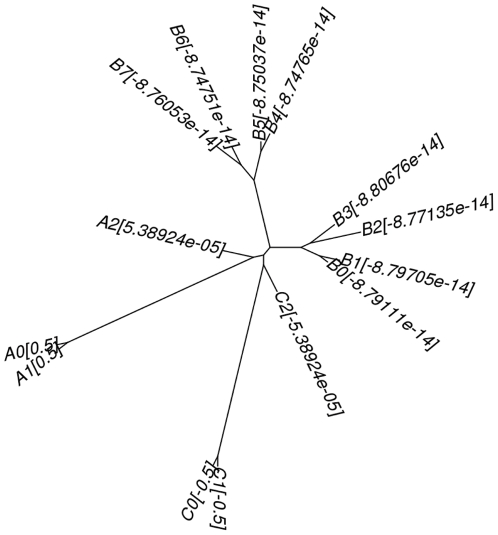
An example illustrating the necessity of a heuristic to avoid placing a large number of genomes in the overlapping region. The tree above depicts the phylogenetic relationships of the input genomes and the eigenvector element computed using 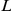 follows a genome name. Two long edges separate A0 and A1 and C0 and C1 from the remaining genomes in the center. The eigenvector elements for the genomes in the center are very small.

Placing too many genomes in the overlapping region is problematic as this increases the redundancy of computing. If there are two long edges separating two small groups of genomes from the remaining genomes, the algorithm described above places a large number of genomes in the overlapping region–genomes in the two small groups have large magnitude eigenvector elements and the remaining genomes have eigenvector elements close to zero. Figure 5 provides an example. A2, B0, B1, B2, B3, B4, B5, B6, B7, and C2 have an eigenvector element with a very small magnitude, but placing these genomes in the overlapping region is problematic.

In this case placing one of the two small groups (A0 and A1 or C0 and C1 in the figure) in one partition and the remaining genomes in the other is a better decomposition. To detect this, *COGNAC* decomposes the two child disks (say Disk 0 and Disk 1) again. If one of the two child disks is decomposed to two non-overlapping disks (say Disk 2 and Disk 3) with one (assume Disk 2) of the two holding all the genomes in the overlapping region of Disk 0 and Disk 1, *COGNAC* places the genomes in Disk 3 in one partition and the remaining genomes in the other instead of placing a large number of genomes in the overlapping region.

#### The Time Complexity and the Space Complexity of the new DCM

To build a binary disk tree, *COGNAC* computes the pairwise distances between the input genomes first and then recursively applies the new DCM. For 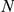 genomes with 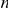 genes per genome, computing the pairwise distances costs 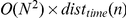 (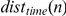 is the time complexity of computing a pairwise distance) operations and 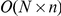 (to store the input data)+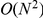 (to store the pairwise distance matrix)+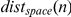 (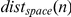 is the space complexity of a pairwise distance computation) space. 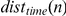 and 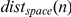 are 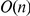 for inversion distance [30]; this leads to the overall time and space complexities of 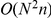 and 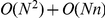, respectively.

In computing a single disk decomposition for a disk with 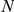 genomes, the time complexity is 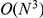 (Intel MKL *dsyevr* eigensolver is the most expensive part in an asymptotic sense) and the space complexity is 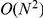. In the theoretical worst case, every disk composition can place only one genome in the two exclusive regions and all the remaining genomes in the overlapping region. In this case, the overall time complexity to build a binary disk tree becomes 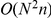
+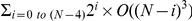 and the space complexity is 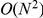
+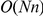
+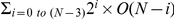 (the last term to store the binary disk tree). In practice, only a small fraction of the genomes in a disk is placed in the overlapping region (and *COGNAC* provides configuration parameters to control the size of the overlapping region such as 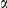 and 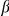). The height of a binary disk tree is 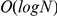 (instead of 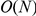 in the worst case) and the size of a disk nearly halves in each decomposition in most practical cases. The 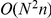 term to compute the pairwise distances takes much longer than the 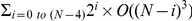 term in our experiments. *COGNAC* builds a binary disk tree in a few seconds or less in all the experiments in this paper while the time spent in processing the binary disk tree is highly input data dependent due to its NP-hard worst case time complexity (evaluating a single topology requires solving multiple NP-hard median problems and *COGNAC* needs to evaluate multiple tree topologies to process the binary disk tree).

### Our Merging Algorithm

Our DCM largely reduces the tree topology search space but does not reconstruct a complete tree. Our merging algorithm fixes details–using the MP principle–to reconstruct a tree for the whole input genomes. To reconstruct a tree of the genomes in a parent disk, our code enumerates candidate tree topologies by merging the sub-topologies of the two child disks; scores those trees by solving median problems; and selects the trees with the lowest parsimony score. Our code has different merging routines for non-overlapping disks and overlapping disks.

#### Merging two non-overlapping disks

To merge two trees in two non-overlapping disks, *COGNAC* first computes the pairwise distances between the genomes–both internal and leaf genomes–in one tree and the genomes in the other tree and finds the pair that minimizes the distance. *COGNAC* enumerates candidate trees by connecting all the neighboring edges of the two vertices. In merging two sub-trees, our algorithm enumerates at most nine topologies unless there is a tie in the minimum distance.

This significantly reduces the number of topologies to be evaluated. Say if a disk with 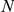 genomes is decomposed to two non-overlapping disks with 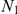 and 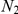 genomes (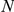 = 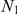
+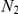), respectively. For 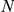 genomes, we need to consider (2
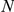
−5)!! topologies with 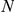 leaf genomes per topology. With the above decomposition, we need to consider 9 or fewer topologies with 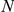 leaf genomes (unless there is a tie in the minimum distance), (2
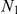
−5)!! topologies with 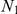 leaf genomes, and (2
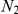
−5)!! topologies with 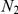 leaf genomes. Assume 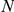, 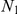, and 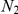 are 10, 5, and 5, respectively. Without the decomposition, we need to consider 2,027,025 topologies with 10 leaf genomes. With the decomposition, we need to consider only 9 topologies with 10 leaf genomes and 2
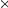
15 topologies with 5 leaf genomes. A recursive application of the new DCM further reduces the candidate tree search space.

#### Merging two overlapping disks

In order to merge two overlapping disks, our algorithm compares the two disks' topology for the genomes in the overlapping region. If there is a conflicting edge, our algorithm collapses the edge similar to [5]. After collapsing all the conflicting edges, our algorithm merges the two sub-trees. Then, our algorithm expands collapsed edges for every possible bifurcating scenario to enumerate candidate tree topologies. As the size of the overlapping region and the number of conflicting edges in the overlapping region increase, the candidate tree search space reduces in a slower rate. *COGNAC* provides configuration parameters (*e.g.* 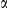 and 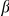) to reduce the size of the overlapping region at the possible risk of lower accuracy.

### Illustrative Examples

Our new DCM not only reduces the number of candidate tree topologies but also excludes erroneous tree topologies which can be selected by original MP methods. The new DCM enables more accurate initial labeling as well. These improve the accuracy of *COGNAC* as a consequence, and this section provides two examples to illustrate this.

**Figure 6:**
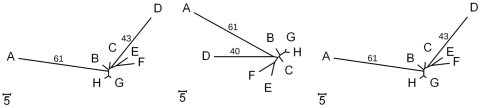
A model tree (left) and two reconstructed trees using an original MP method (the GRAPPA method) (center) and *COGNAC* (right). Numbers on top of edges are edge lengths.

Figure 6 depicts a model tree used to generate test data and two reconstructed trees using an original MP method (the GRAPPA method) and *COGNAC* . For the result, the length of an edge is identical to the inversion distance between the two genomes in each side of the edge–this is smaller than the number of inversions that happened in the edge if there is a parallel mutation within the edge. Each genome has 150 genes and inversion lengths used to generate test data follow the gamma distribution (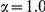 and 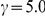). The original MP method returns four erroneous trees, and the figure depicts one of the four. The tree joins two long edges together, and this topology is vulnerable to long branch attraction. *COGNAC* does not include the topology in the search space. Our DCM first places 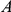 in one partition and the remaining genomes in the other partition. After reconstructing a tree for the genomes in the other partition, *COGNAC* enumerates candidate trees by first finding the genome closest to A–the immediate ancestor of B is closest to A–and connecting A to the genome's three neighboring edges. No tree in the search space joins the external edges to A and D. Our DCM not only reduces the search space but also improves the accuracy by excluding many erroneous trees from the search space.

**Figure 7:**
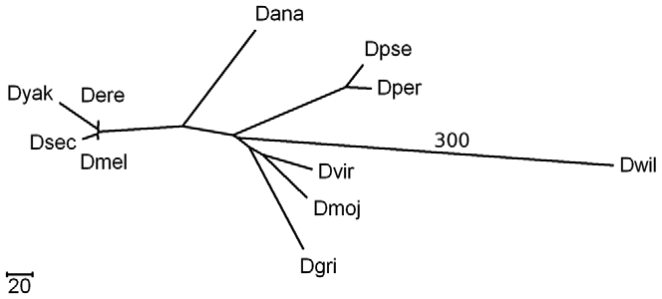
A model tree used to demonstrate the superiority of *COGNAC* in initializing internal nodes. The figure depicts a model tree adopted from a biology paper [20].

The new DCM often leads to better initialization of internal genomes as well. Figure 7 depicts a model tree adopted from Figure 8 of Bhutkar *et al.* 's paper [20]. The paper does not provide the length of the external edge to *D. willistoni* (Dwil in Figure 7) and we set the edge length to a large number. We also scale down the edge lengths to experiment with significantly non-additive trees while limiting the execution time. We set the number of genes in each gene order data to 2000 first and reduce the number by 200 till *COGNAC* or the original MP method does not finish within a practical time limit. For 1200 genes, the original MP method runs for 7.3 hours and returns two erroneous trees (one false positive and two false negatives for one tree and two false positives and two false negatives for the other tree). *COGNAC* finishes in 40 milliseconds and retrieves the 100% accurate topology. The original MP method does not finish within a practical limit for 1000 or less genes. *COGNAC* reconstructs the 100% accurate topology even for 800 genes (in 2.2 hours).

**Figure 8:**
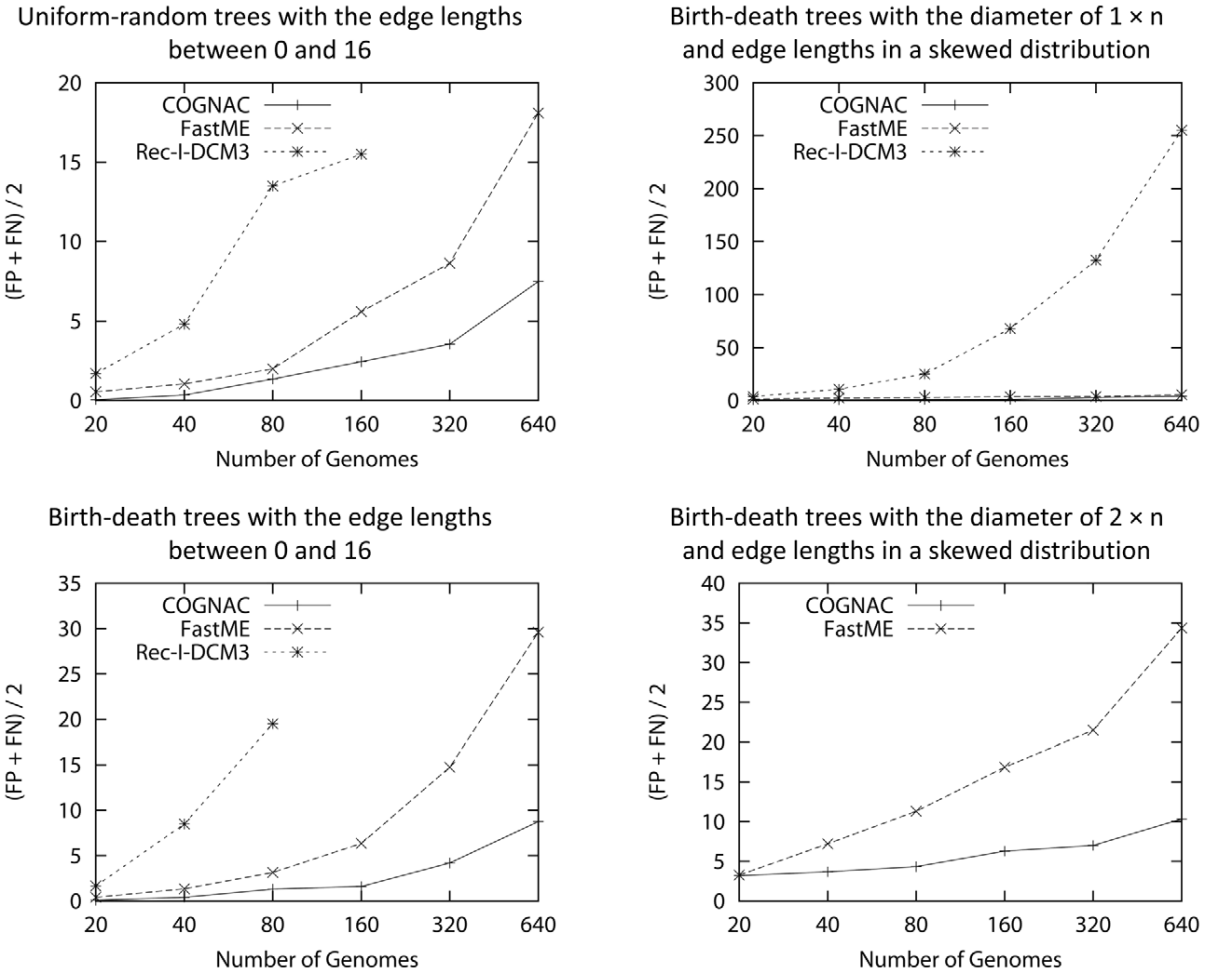
A summary of the experimental results. The figures plot (FP+FN)/2 (FP is the number of false positives and FN is the number of false negatives) for a varying number of genomes (20, 40, 80, 160, 320, 640). The numbers are the average of the finished computations. Missing points indicate that no computation finished within 24 hours. See Tables 1, 2, 3, and 4 for additional details.

This is mainly due to better initialization. If there are only a small number of evolutionary events among a set of genomes, it is often not very difficult to accurately reconstruct the evolutionary events and the ancestral genomes. If there are a large number of events among a set of genomes, reconstructing the evolutionary events and the ancestral genomes becomes much more challenging. With the new DCM, we can initialize internal genomes using closer genomes, which is generally more accurate than initializing internal genomes using distant genomes. Without the new DCM, it is difficult to find the best order to initialize the internal genomes, and the original MP method initializes the internal genomes using the three nearest leaf genomes. For example, to initialize the immediate ancestor of Dwil, the GRAPPA method finds the median genome of Dwil and two more leaf genomes (*e.g.* Dmel and Dvir). *COGNAC* first places Dwil in one partition and the remaining genomes in the other partition. To merge the two partitions, *COGNAC* finds the genome closest to Dwil and breaks the genome's three neighboring edges to connect Dwil. At this time, all the internal genomes except for the immediate ancestor of Dwil are already initialized. *COGNAC* can initialize the immediate ancestor of Dwil by finding the median genome of Dwil and the Dwil's immediate ancestor's two neighboring internal genomes. The new DCM forces to initialize the internal genomes in a desirable order which is difficult to find in the case of the original MP method.

## Results and Discussion

### Experimental Setup

We test our new method using gene order data generated using various simulators and assuming inversion distance. We run our code on a system with two 2.4 GHz quad-core Intel Nehalem-EP processors (E5330) and 12 GB DRAM. *COGNAC* is multi-threaded (using Intel Thread Building Block), but we configure *COGNAC* to use only one core in the system to compare with other single-threaded software packages.

We generate model trees using simulators used in Tang and Moret's work [13] and Lin *et al.* 's work [14]. Tang and Moret [13] ran tests using uniform-random trees and birth-death trees in their DCM-GRAPPA paper (for birth-death trees, they used the r8s software package [15] developed by Sanderson). They generated trees with identical edge lengths (all the edges in a single tree have an identical edge length) of 2, 4, and 8. This is less realistic, and it is also easy to reconstruct the model tree if all the edges in a model tree have an identical edge length. We set an edge length by randomly sampling an integer number between 0 and 16 instead. We also use model trees with skewed edge length distributions (a small number of long edges and many short edges) using the simulator introduced in [14]. The simulator generates birth-death trees using the R statistics package first (using the birth-rate of 0.001 and the death rate of 0) and modifies the edge lengths to generate non-ultrametric trees. To modify an edge length, the simulator randomly samples a floating point number (say 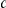) between −2 and 2 and multiplies 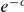 to the edge length. After modifying all the edge lengths, the simulator uniformly scales the edge lengths to generate a tree with a specific diameter. We use 1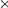 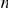 (the number of genes in a genome, 100 in our experiments) and 2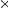 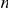 as diameters of the generated trees. We generate leaf genomes by applying inversions to a common ancestral genome with 100 genes. The number of inversions applied in each edge is identical to the length of an edge, and the inversion lengths follow the uniform distribution. We use the generated leaf genomes as input data.

There are several software packages to reconstruct a phylogenetic tree for a large number of genomes using gene order data. FastME is a widely used program based on use of distance methods. Distance methods' performance depends on the quality of the input distance matrix. We use GRAPPA 's EDE to correct inversion distance to better estimate the true number of inversions happened in the evolutionary history and feed the corrected distances as FastME input data. We also test with uncorrected distances and CDCJ [31] corrected distances, but GRAPPA 's EDE produces the best results. We present the results with the EDE in this paper. FastME has several options, and we use the NJ initialization, BNNI, and SPR as this combination produces the most accurate trees in our experiments.

DCM2 and Rec-I-DCM3 are options for MP methods. Tang *et al.* [13] presented experimental results using the combination of DCM2 and GRAPPA in their DCM-GRAPPA paper. They used trees with identical edge lengths as mentioned in the previous paragraph, and DCM-GRAPPA ran for ten hours to two days and reconstructed trees (640 leaf genomes) with approximately 10 false positives (when edge length is 2), 1 false positive (when edge length is 4), and close to 0 false positive (when edge length is 8) on average. *COGNAC* reconstructs nearly 100% accurate topologies in seconds to minutes for such trees. It is easy to reconstruct an accurate tree if all the edges in a model tree have an equal length, and as DCM-GRAPPA ran much slower than *COGNAC* to return less accurate trees even in this case, we do not further compare *COGNAC* with DCM-GRAPPA . Instead, we compare *COGNAC* with the combination of the state of the art DCM (Rec-I-DCM3) and GRAPPA . We use the FastME output trees as a guide tree for Rec-I-DCM3 . We set the maximum subset size to 8 and the iteration count to 3 in reconstructing trees using Rec-I-DCM3 . We set maximum execution time to 24 hours, and if the execution time exceeds 24 hours, we mark the computation as unfinished.

### Experimental Results and Analysis

Figure 8 summarizes experimental results for uniform-random trees with the edge lengths between 0 and 16, birth-death trees with the edge lengths between 0 and 16, birth-death trees with a skewed edge length distribution and the diameter of 1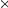 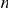, and birth-death trees with a skewed edge length distribution and the diameter of 2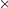 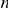, respectively. See Tables 1, 2, 3, and 4 for additional details. FastME in the figure and tables represents the combination of GRAPPA 's EDE and FastME using the options described above. Rec-I-DCM3 in the figure and tables represents the combination of Rec-I-DCM3 and GRAPPA using the FastME output tree as a guide tree. If *COGNAC* returns more than one tree, we randomly select one of the returned trees. FastME returns a tree with edge lengths in a floating point number. If an edge is shorter than 0.5, we consider the edge as a zero length edge in computing the topological accuracy. We count errors for only internal edges–assigning length zero for a non-zero external edge in the model tree or assigning non-zero length to a zero-length external edge in the model tree does not increase the number of false positives or false negatives.

**Table 1:** Experimental results for uniform-random trees with the edge lengths between 0 and 16.

	*COGNAC*				FastME				Rec-I-DCM3			
*N*	finished	FP	FN	time	finished	FP	FN	time	finished	FP	FN	time
20	10	0	0.1	0.17 s	10	0.8	0.3	0.11 s	10	1	2.4	51 m
40	10	0.5	0.2	0.51 s	10	1.6	0.5	0.099 s	8	2.88	6.75	6.8 h
80	10	1.8	0.9	4.9 s	10	3.1	0.9	0.13 s	2	8.5	18.5	3.1 h
160	10	2.8	2.1	19 s	10	7.7	3.5	0.26 s	1	9	22	7.0 h
320	10	4.1	3	1.1 m	10	11.5	5.8	1.8 s	0	N/A	N/A	N/A
640	10	8.4	6.6	3.4 m	10	25.1	11.1	46 s	0	N/A	N/A	N/A

We generate 10 model trees for a given number of genomes (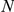). The number of false positives (FP), the number of false negatives (FN), and the execution time (time) in a cell are the average of the finished computations (finished: the number of finished computations within 24 hours) out of 10 trials using 10 different model trees. 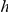, 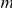, and 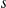 in the tables are hours, minutes, and seconds, respectively.

**Table 2:** Experimental results for birth-death trees with the edge lengths between 0 and 16.

	*COGNAC*				FastME				Rec-I-DCM3			
*N*	finished	FP	FN	time	finished	FP	FN	time	finished	FP	FN	time
20	10	0.2	0	0.16 s	10	0.7	0.1	0.23 s	10	0.9	2.4	1.5 h
40	10	0.5	0.3	0.75 s	10	1.8	0.9	0.22 s	5	4.8	12.2	8.7 h
80	10	1.6	1	7.9 s	10	4.4	1.9	0.23 s	1	15	24	23 h
160	10	2.2	1	23 s	10	8.3	4.4	0.66 s	0	N/A	N/A	N/A
320	10	5.4	3	1.3 m	10	19	10.5	3.3 s	0	N/A	N/A	N/A
640	10	10.3	7.2	4.8 m	10	37	22.2	25 s	0	N/A	N/A	N/A

We generate 10 model trees for a given number of genomes (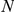). The number of false positives (FP), the number of false negatives (FN), and the execution time (time) in a cell are the average of the finished computations (finished: the number of finished computations within 24 hours) out of 10 trials using 10 different model trees. 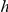, 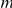, and 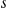 in the tables are hours, minutes, and seconds, respectively.

**Table 3:** Experimental results for birth-death trees with the diameter of 1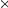 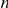 and edge lengths in a skewed distribution.

	*COGNAC*				FastME				Rec-I-DCM3			
*N*	finished	FP	FN	time	finished	FP	FN	time	finished	FP	FN	time
20	10	0.7	0.7	0.18 s	10	1.4	1.4	0.16 s	9	2.33	5.11	1.3 h
40	10	0.5	0.9	0.21 s	10	1.4	3.2	0.23 s	9	5.33	16.2	5.5 h
80	10	0.7	1.1	0.91 s	10	2	3.7	0.27 s	9	13.6	36.7	2.5 h
160	10	0.9	1.3	2.4 s	10	2.5	4.7	0.57 s	7	42.7	93.1	6.0 h
320	10	2.1	3.5	19 s	10	2.1	5.4	2.0 s	7	82.1	183	8.9 h
640	10	2.6	5.7	2.9 m	10	2.8	8.3	13 s	6	155	355	6.2 h

We generate 10 model trees for a given number of genomes (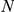). The number of false positives (FP), the number of false negatives (FN), and the execution time (time) in a cell are the average of the finished computations (finished: the number of finished computations within 24 hours) out of 10 trials using 10 different model trees. 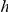, 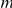, and 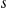 in the tables are hours, minutes, and seconds, respectively. 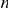 is the number of genes in a genome, which is 100 in our experiments.

**Table 4:** Experimental results for birth-death trees with the diameter of 2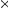 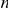 and edge lengths in a skewed distribution.

	*COGNAC*				FastME				Rec-I-DCM3			
*N*	finished	FP	FN	time	finished	FP	FN	time	finished	FP	FN	time
20	5	3.2	3.2	1.2 h	10	3.2	3.3	0.25 s	0	N/A	N/A	N/A
40	6	3.5	3.83	52 m	10	6.8	7.6	0.24 s	0	N/A	N/A	N/A
80	6	3.67	5	19 m	10	10.6	12	0.27 s	0	N/A	N/A	N/A
160	7	5.57	7	34 m	10	15.1	18.6	0.69 s	0	N/A	N/A	N/A
320	5	6.4	7.6	14 m	10	18	25	3.4 s	0	N/A	N/A	N/A
640	6	7.83	12.8	14 m	10	25.8	42.9	28 s	0	N/A	N/A	N/A

We generate 10 model trees for a given number of genomes (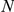). The number of false positives (FP), the number of false negatives (FN), and the execution time (time) in a cell are the average of the finished computations (finished: the number of finished computations within 24 hours) out of 10 trials using 10 different model trees. 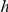, 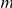, and 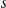 in the tables are hours, minutes, and seconds, respectively. 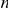 is the number of genes in a genome, which is 100 in our experiments.

We generate 10 model trees for each test case. The number of false positives (FP), the number of false negatives (FN), and the execution time (time) in the figure and tables are the average of the finished computations out of 10 trials using 10 different model trees. 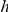, 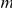, and 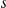 in the tables are hours, minutes, and seconds, respectively.

*COGNAC* clearly outperforms FastME and Rec-I-DCM3 in accuracy. *COGNAC* also consistently reconstructs accurate trees regardless of model tree types. In contrast, FastME is less accurate for birth-death trees than uniform-random trees, and FastME 's accuracy drops significantly for birth-death trees with a larger diameter and a large number of genomes. Rec-I-DCM3 fails to improve the accuracy of the input guide tree especially for trees with a skewed edge length distribution. In reconstructing trees for 640 input genomes, *COGNAC* reconstrus a tree with 15 false positives and 13 false negatives in the worst case (this happens with a birth-death tree with the edge lengths between 0 and 16), while FastME returns a tree with 34 false positives and 64 false negatives (this is for a birth-death tree with the diameter of 2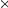 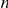 and edge lengths in a skewed distribution) and Rec-I-DCM3 retunrs a tree with 166 false positives and 384 false negatives (this is for a birth-death tree with the diameter of 1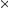 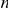 and edge lengths in a skewed distribution, Rec-I-DCM3 fails to output any tree for a model tree with the diameter of 2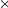 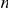 within the time limitation) in the worst case; this shows the robustness of *COGNAC* .

*COGNAC* is much faster than Rec-I-DCM3 (both are based on the MP principle) but is slower than FastME which is a distance method. The most expensive routine in both *COGNAC* and Rec-I-DCM3 (in combination with GRAPPA) is a median solver. A median solver solves a median problem which finds a median genome of three genomes. The execution time to find a median genome highly depends on the distances between the three genomes. If the genomes become more distant than a certain threshold, the execution time increases very fast [32] (note that the worst case time complexity of Caprara's median solver–both *COGNAC* and GRAPPA use Caprara's median solver–is NP-hard). *COGNAC* 's DCM places a smaller number of genomes–often 0–in the overlapping region. *COGNAC* also decomposes disks till three or fewer genomes are left while Rec-I-DCM3 requires the minimum disk size larger than a certain threshold to be accurate. Rec-I-DCM3 requires multiple iterations as well. *COGNAC* needs to solve fewer median problems for closer genomes as a consequence, and this explains *COGNAC* 's faster execution time.

MP methods for gene order data generally run multiple orders of magnitude slower than distance methods. Even though *COGNAC* runs slower than FastME , the difference is much less than the difference between typical MP and distance methods using gene order data. *COGNAC* is reasonably fast in most cases but becomes very slow if *COGNAC* needs to find a median genome of three distant genomes (*COGNAC* 's execution time to reconstruct a phylogenetic tree for 640 genomes varies from less than 30 seconds to over 24 hours); this is not an artifact of our newly developed DCM but a shortcoming of the median algorithm used in *COGNAC* . There are faster median solvers in the literature– *e.g.* [32]–[34] for inversion median and [35] for DCJ median, but even those become very slow for very distant genomes. This necessitates the development of faster median solvers.

### Conclusions and Future Work

We design a new DCM based on the spectral method and also develop the *COGNAC* software package which uses the new DCM. *COGNAC* demonstrates both high accuracy and fast execution time, and our new DCM plays a significant role in *COGNAC* 's superior accuracy and fast execution time. The new DCM significantly reduces the candidate tree search space. If the new DCM excludes a tree that best captures the phylogenetic relationships from the search space, however, this significantly lowers the value of the new DCM. *In obtaining all the results presented in the previous section, our new DCM never returns an incompatible decomposition; thus, the new DCM never excludes the model tree topology from the search space.* We perform intensive tests to find a case our new DCM returns an incompatible decomposition–we run experiments using both uniform-random trees and birth-death trees with various edge length distributions (*e.g.* the average edge length of 2, 4, 8 and using various deviations from the average length) and also using manually created trees to challenge our DCM. We find our new DCM sometimes returns an incompatible decomposition–though very rarely–if the model tree of the genomes to decompose has only short edges (*e.g.* if every edge has the length of 2). Even in this case, most of the incompatible decompositions are recoverable and actually recovered (if the tree has only short edges, most reasonable reconstruction methods find a very accurate tree). Our new DCM returns an irrecoverable decomposition in extremely rare cases; when the input genomes consists of multiple highly distant groups (if such groups are very distant and all the phylogenetic signals between the groups are lost, no method can reconstruct the phylogeny) or statistically very unlikely events happen (*e.g.* if the model tree has only a small number of short edges but the tree has many parallel mutations). All the errors in the experimental results obtained using *COGNAC* in this paper are introduced in the merging step.

The new DCM significantly improves the speed and accuracy of *COGNAC* , but there are still remaining challenges to reconstruct accurate phylogenetic trees within a practical time limit–most importantly, finding a more accurate and flexible distance metric (than inversion distance) and designing a median solver with a bounded complexity. Especially *COGNAC* currently supports only inversion and assigns the weight of 1 to every inversion regardless of their length. Ideally phylogenetic tree reconstruction software needs to support various types of chromosome-level mutations (such as inversion, translocation, transposition, fusion, fission, insertion, deletion, and duplication). Most currently available distance metrics also assign a same weight for a same type of mutations–with an exception of [36]. This is more due to computational efficiency rather than biological fidelity. A flexible distance metric that allows users to assign different weights based on the type and length of a mutation and possibly the significance of the genes (or other genetic markers) involved in the mutational event is highly desirable. The newly designed DCM significantly reduces the number of median problems to be solved and pairwise distances to be computed while the use of a large scale supercomputer and high-performance computing techniques can provide a much larger computing capacity; this enables us to adopt a computationally more expensive but more flexible and biologically justifiable distance metric. We will focus on addressing remaining problems by adopting new effective algorithms designed by many other researchers in the community, designing new algorithms by ourselves, and using parallel computers (including a leadership scale supercomputer) and performance optimization techniques. All these combined, *COGNAC* will evolve towards a highly flexible and powerful tool to assist biologists to better understand the evolutionary history of living species using gene order data.
